# Nutrition risk stratification and metabolic disorders in patients on parenteral nutrition

**DOI:** 10.1590/1980-220X-REEUSP-2024-0274en

**Published:** 2025-04-11

**Authors:** Rodrigo Luis da Silveira Silva, Ranna Rayssa Leal dos Santos, Helder Cardoso Tavares, Adrielle Cavalcanti de Pontes Araújo, Ulisses Ramos Montarroyos, Ana Célia Oliveira dos Santos

**Affiliations:** 1 Universidade de Pernambuco, Faculdade de Ciências Médicas, Recife, PE, Brazil.; 2 Universidade de Pernambuco, Instituto de Ciências Biológicas, Recife, PE, Brazil.; 3 Universidade de Pernambuco, Hospital Universitário Oswaldo Cruz, Recife, PE, Brazil.

**Keywords:** Nutrition Assessment, Parenteral Nutrition, Sodium, Magnesium, Avaliação Nutricional, Nutrição Parenteral, Sódio, Magnésio

## Abstract

**Objective:**

To evaluate the association between metabolic disorders and death in patients receiving Parenteral Nutrition (PN), according to a proposal for stratification into nutri­tional risk groups, using the Nutritional Risk Screening, 2002 (NRS-2002).

**Method:**

A cohort with 84 patients receiving PN, with an NRS-2002 score of ≥3, classified into two subgroups: moderate risk (NRS 3-4) and severe risk (NRS 5-7). Secondary data from medical and nutritional records were used. The outcome variable was “presence of metabolic disorders and death”; and exposures: moderate and severe nutritional risk. Descriptive, bivariate analyses and logistic regression models were performed.

**Results:**

No difference was observed between the groups in terms of death. The adjusted RR (95% CI) for sodium disorders was 4.88 (1.5–16.2) (p = 0.009), and for magnesium disorders, 7.58 (2.33–24.6) (p = 0.001), being higher in the severe risk group.

**Conclusion:**

The stratification of the nutritional risk range into moderate and severe risk was able to identify patients at higher risk of developing metabolic disorders, especially sodium and magnesium ones.

## RESUMO

**Objetivo:**

Avaliar a associação entre distúrbios metabólicos e morte em pacientes que recebem Nutrição Parenteral (NP), de acordo com uma proposta de estratificação em grupos de risco nutricional, utilizando o Nutritional Risk Screening, 2002 (NRS-2002).

**Método:**

Uma coorte com 84 pacientes recebendo NP, com um escore NRS-2002 ≥ 3, classificados em dois subgrupos: risco moderado (NRS 3-4) e risco grave (NRS 5-7). Foram utilizados dados secundários de registros médicos e nutricionais. As variáveis de desfecho foram “presença de distúrbios metabólicos e óbito” e as de exposição, “risco nutricional moderado e grave”. Foram realizadas análises descritivas, bivariadas e modelos de regressão logística.

**Resultados:**

Não foi observada diferença entre os grupos em termos de óbito. O RR ajustado (IC ٩٥٪) para distúrbios de sódio, ٤,٨٨ (١,٥–16,2) (p = 0,009), e para distúrbios de magnésio, 7,58 (2,33-24,6) (p = 0,001), foi maior no grupo de risco grave.

**Conclusão:**

A estratificação da faixa de risco nutricional em risco moderado e grave foi capaz de identificar pacientes com maior risco de desenvolver distúrbios metabólicos, especialmente de sódio e magnésio.

## INTRODUCTION

Hospitalized patients may present malnutrition at the time of hospital admission or develop it during hospitalization^(1)^. In Latin American countries, 40 to 60% of hospitalized adult patients present with malnutrition at the time of admission^(2)^. This high prevalence is associated with longer hospital stays, major complications (pressure injuries, infections, falls), a higher frequency of readmissions and hospital costs, in addition to higher mortality rates^(2,3)^. There are several validated tools that may be used for nutritional risk screening to estimate the probability of either a better or worse prognosis through risk factors associated with malnutrition^(4)^.

The Nutritional Risk Screening 2002 (NRS 2002) is one of the most commonly used tools in clinical practice, carried out through a quick and simple process, led by the hospital admission team (doctor, nurse or dietitian), includes domains related to nutritional status and disease severity, and adds one extra point if the patient is aged over 70^(5)^. The nutritional status score (NSS) mainly assesses the percentage of weight loss over time, the body mass index (BMI), and the recent reduction in food consumption. The disease severity score (DSS) is based on the impact caused by a diagnosis or clinical condition on a patient’s nutritional requirements^(5)^.

It has been reported that patients who are at nutritional risk (NRS ≥ 3) have longer hospital stays and present surgical complications^(4,6)^, higher and earlier mortality rates^(7)^, and higher hospital costs^(8)^ when compared to patients without nutritional risk. It has been demonstrated that, in medical inpatients, for every 1-point increase in the NRS score, a significant increase in mortality was observed at 30 and 180 days, as well as longer hospital stay (and a poorer functional prognosis at 180 days)^(9)^. This result suggests that an interpretation of the NRS 2002 result through the current classification (no risk < 3 and with risk ≥ 3) may underestimate the severity of the nutritional condition and its associated complications and may also interfere with defining the level of individualized nutritional assistance for each patient.

Parenteral nutrition (PN) is an expensive, complex therapy, often used when patients present with clinical conditions associated with nutritional risk or malnutrition, which may contribute to the appearance of serious metabolic and infectious complications, increased hospitalization time and morbidity when it is not properly administered or prescribed and must be continuously assessed and monitored^(10,11)^. Considering that patients on PN are either malnourished or at nutritional risk and that these conditions may aggravate their prognosis, this study has aimed to assess the association between the risk of metabolic disorders and death, according to a new proposal for stratification into nutritional risk groups (moderate and severe).

It is worth noting that the new stratification proposal does not aim to change the elements that make up the NRS 2002, so it can be used with the same population and scope as the original study, and can be applied to hospitalized adult and older patients, regardless of the route used for the patient’s nutrition (oral, enteral or parenteral). However, it suggests a new way of interpreting the results, improving the clinical applicability of this tool.

## METHOD

### Study Design, Period, and Location

The present study is described in accordance with the checklist on the STROBE statement^(12)^. This was a retrospective observational cohort study at a public university hospital, a referral center for infectious and parasitic diseases, carried out from June 2020 to August 2022.

### Population and Sample: Inclusion and Exclusion Criteria

The population consisted of 84 adult patients receiving Total Parenteral Nutrition (TPN) who were hospitalized at the study site. The sample was for convenience and consisted of patients hospitalized during the study period and who used PN as an exclusive route for nutrition. Patients included were ≥ 18 years old, with nutritional risk (NRS ≥ 3), receiving central or peripheral TPN, admitted to the wards and intensive care units. The NRS 2002 was carried out when patients were admitted to have nutritional risk identified. The screening initially investigates whether the BMI is less than 20.5 kg/m^2^, whether the patient has lost weight in the last 3 months, whether there has been a reduction in food intake in the last week, or whether the patient is seriously ill. If any of the answers were affirmative, the screening went on to the next stage, where the deterioration in nutritional status and the severity of the illness or degree of stress were measured. Based on the results found, the patient was classified as being at nutritional risk if the score was ≥ 3. Patients who received parenteral nutrition for less than 24 hours and those who had metabolic or electrolyte disturbances that could not be corrected before starting PN, were excluded.

### Study Protocol

Data were collected by those responsible for the study of the patient’s medical records, nutritional monitoring form, and clinical follow-up. The following data were collected: sex, age, presence of comorbidities, place of hospital admission, length of hospital stay, time in days to initiate PN and the total time of use, the type of venous access used. Early PN was defined when its onset occurred within 48 hours of the patient’s hospitalization, the surgical or clinical event that indicated PN. The observed outcomes were the presence of glycemic and electrolyte disorders (Na, K, P and Mg) while using the PN exclusively, and the outcome (death/discharge) until the end of the PN. There was no follow-up to assess the recurrence of metabolic disorders after patients were discharged. Laboratory tests were performed daily and requested according to the protocol established at the hospital, adapted according to Worthington et al.^(13)^.

Among the patients included in the study, based on the nutritional risk screening (NRS 2002), 2 subgroups were formed: moderate risk (score 3-4) and severe risk (score 5-7). The reasoning used to stratify the groups was based on what has already been shown in the literature, that for every 1-point increase in the nutritional risk score, the risk of developing complications increases. We therefore decided to divide the nutritional risk range (score 3 to 7) into two strata (3-4) and (5-7).

Anthropometric measurements were taken to identify weight and height to calculate the Body Mass Index (BMI), one of the criteria used in the initial phase of NRS 2002. The current height and weight of patients who were able to walk were measured, and the measurements of those who were unable to walk were estimated according to Chumlea et al.^(14,15)^. To estimate nutritional needs, the weight measured in the anthropometric assessment was used; however, in obese patients, the adjusted weight was used^(16)^, and for those with anasarca, the average weight according to the body mass index (BMI) was used for adults and the older people^(17,18)^.

Patients were classified, according to the hospital protocol, in critical if they were hospitalized in an intensive care unit, with or at risk of developing vital organ failure, and in need of continuous surveillance and life support (ventilatory, hemodynamic or renal replacement therapy). Patients were considered stable if they were admitted to the ward without the need for continuous surveillance or ventilatory or hemodynamic support. Indications for PN use followed ASPEN guidelines^(13)^.

Glucose and electrolyte disorders were considered as the presence of plasma levels above or below the reference range used by the institution’s laboratory: glycemia: stable patients (60–99 mg/dl) and critically ill patients (60–180 mg/dl); sodium (135–148 mEq/l); potassium (3.5–5.3 mEq/l); phosphorus (2.7–4.5 mg/dl); magnesium (1.46–2.2 mg/dl); AST (10–50 U/l); ALT (10–50 U/l).

The protein and caloric intake of stable patients was considered adequate when contained within the range of 1.2 to 2.0g of protein/kg/day and 25 to 35kcal/kg/day, respectively^(19,20)^. In critically ill patients, protein intake of 1.5 to 2.0g of protein/kg/day and caloric intake of 15 to 20 Kcal/kg/day until the 4^th^ day of PN, and of 25 to 30 Kcal/kg/day from the 5^th^ day in patients in the recovery phase^(20)^ were considered adequate. The percentage of caloric and protein adequacy in relation to the established goals was calculated, considering a daily caloric and protein intake of at least 70% of the estimated nutritional needs as being adequate.

The PN was prescribed taking into account the clinical and nutritional diagnosis of each patient, respecting the limits established in the nutritional therapy guidelines for liquids, macronutrients and micronutrients, so as not to induce metabolic disturbances caused by inadequate nutrient administration: glucose (4-5 mg/kg/min), lipids (<1g/kg/day), liquids (30–40ml/day), calcium (10–15 mEq/day), magnesium (8–20 mEq/day), phosphorus (20–40 mmol/day), sodium (1–2 mEq/kg/day), potassium (1-2 mEq/kg/day), as well as recommended daily doses of vitamins and trace elements^(21)^.

All patients received individualized PN bags, prescribed and monitored by a multidisciplinary team of physicians, registered dietitians, nurses, and pharmacists. The PN bags were manipulated according to the daily nutritional prescription and administered within 24 hours through a continuous infusion pump. For the evolution of the nutritional behavior, the protocol established by the institution was applied, where the patients began with 30% of their estimated nutritional needs (with at least 100g of glucose/day), progressing to 50%, 70% and reaching 100% after the 5th day, if the patient had no complications.

### Analysis of Results and Statistics

Data was analyzed using the STATA IC 14.2 (STATA Corporation). Categorical variables were described in absolute and relative frequency measures, while numeric variables were described in median and interquartile ranges (P_25_ – P_75_) since they did not present normal distribution. To test the association between binary categorical variables, Pearson’s chi-square test (2×2) was used. Logistic regression analysis was applied to estimate the relative risk (RR) and respective confidence intervals (95% CI) in the association of outcomes with nutritional risk groups. To adjust for confounding factors, the criterion adopted was significance of up to 10% (p < 0.1) in the bivariate analysis with nutritional risk. According to this criterion, the RR was adjusted for BMI, type of access, and length of stay. The RR was presented with and without multivariate adjustment to show the effect of these variables on the association between nutritional risk and outcomes. The adopted statistical significance was 5% for all analyses.

### Statement of Ethics

The study protocol was conducted in accordance with national and international ethical guidelines and approved by the Research Ethics Committee of the Hospital Universitario Oswaldo Cruz (HUOC)/ Pronto Socorro Cardiológico Universitario de Pernambuco Prof. Luiz Tavares (HUOC/PROCAPE Hospital Complex), approval number [4.432.953]. Patient consent was not required because it was retrospective research that used data that had already been routinely collected on nutritional monitoring forms, patient records, or electronic databases. Furthermore, the data was analyzed and presented anonymously and aggregated, not allowing the identification of research participants.

## RESULTS

Ninety patients were initially recruited, but 6 were excluded for receiving PN for a period less than 24 hours due to death or clinical complications, thereby totaling 84 patients. There were no other losses or exclusions, as the other exclusion criterion was the presence of metabolic or electrolyte disorders that could not be corrected before starting PN, which is rare, as the service has a protocol for correcting metabolic and electrolyte disorders before starting PN.

The study population presented a median length of hospital stay of 21.5 days and was mostly represented by older males, hospitalized in surgical wards (67.9%), with no comorbidities (52.4%), and with a median age of 61.5 years and a BMI of 20.4 kg/m^2^. The PN was mainly administered through central venous access (79.8%) and PN median time was 9 days. Furthermore, it was also observed that 71.4% of patients initiated parenteral nutrition early. More than 80% of the patients presented caloric and protein adequacy above 70% of the estimated nutritional needs. Through the NRS 2002, it was verified that most patients presented a nutritional risk score equal to 5 (42.9%) and that this result was composed mainly of severe depletion of the patients’ nutritional status (Score 3 = 53.6%) and moderate severity of the disease (Score 2 = 65.5%, shown in Table 1).

**Table 1:** Characteristics of patients receiving parenteral nutrition – Recife, PE, Brazil, 2022.

Characteristics	n [%]/median/mean
**Sex**	
Male	44 [52.4%]
**Age range**	
Adult (< 60 years)	40 [47.6%]
Older people (≥ 60 years)	44 [52.4%]
**BMI (kg/m_2_)**	20.4 [17.9–26.9]
**With Comorbidities**	40 [47.6%]
**Place of stay in hospital**	
ICU	16 [19.0%]
Medical Ward	11 [13.1%]
Surgical Ward	67 [67.9%]
**Type of venous access**	
Peripheral	17 [20.2%]
Central	67 [79.8%]
**Days of hospital stay**	21.5 [13–32]
**Days on PN**	9 [6–18]
**Early PN**	60 [71.4%]
**NRS, 2002 score composition**	
***Nutritional status***	
Score 0	8 [9.5%]
Score 1	11 [13.1%]
Score 2	20 [23.8%]
Score 3	45 [53.6%]
***Disease severity***	
Score 0	1 [1.2%]
Score 1	15 [17.9%]
Score 2	55 [65.5%]
Score 3	13 [15.5%]
***Age (> 70 years)***	
Score 0	68 [81%]
Score 1	16 [19%]
**Adequate calories**	92.5 [±14.9]
> 70%	74 [88.1%]
≤ 70%	10 [11.9%]
**Adequate protein**	93.8 [±14.1]
> 70%	76 [90.5%]
≤ 70%	8 [9.5%]
**Glucose disorder**	72 [85.7%]
**Sodium disorder**	55 [65.5%]
**Potassium disorder**	55 [65.5%]
**Phosphorus disorder**	53 [63.1%]
**Magnesium disorder**	36 [42.9%]

BMI: Body Mass Index; PN: Parenteral Nutrition; NRS 2002: Nutritional Risk Screening 2002; Early: ≤ 48h after hospitalization/surgery.

To verify whether there was a difference between the moderate and severe risk groups, an association was initially made between the analyzed cofactors and the respective groups. No differences were observed between groups in most variables, apart from BMI, which was lower in the severe nutritional risk group (p = 0.001) (shown in Table 2).

**Table 2:** Association of analyzed variables in relation to nutritional risk by the NRS, 2002 score – Recife, PE, Brazil, 2022.

Variables	Moderate risk [n = 40]	Severe risk [n = 44]	p*
Sex			
Female	18 [45%]	22 [50%]	0.647
Male	22 [55%]	22 [50%]	
BMI (kg/m_2_)	24.5 [19.9–28.7]	18.7 [16.2–22.5]	0.001
Comorbidities			
No	22 [55%]	22 [50%]	0.647
Yes	18 [45%]	22 [50%]	
Type of venous access			
Peripheral	5 [12.5%]	12 [27.3%]	0.092
Central	35 [87.5%]	32 [72.7%]	
Days of hospital stay			
≤ 21 days	16 [40%]	26 [59.1%]	0.081
> 21 days	24 [60%]	18 [40.9%]	
Days on PN			
≤ 9 days	18 [45%]	26 [59.1%]	0.197
> 9 days	22 [55%]	18 [40.9%]	
Early PN			
No	11 [27.5%]	13 [29.5%]	0.836
Yes	29 [72.5%]	31 [70.5%]	
Adequate calories			
> 70%	6 [15%]	4 [9.1%]	0.404
≤ 70%	34 [85%]	40 [90.9%]	
Adequate protein			
> 70%	4 [10%]	4 [9.1%]	0.887
≤ 70%	36 [90%]	40 [90.9%]	

BMI: Body Mass Index; PN: Parenteral Nutrition; NRS 2002: Nutritional Risk Screening 2002; Early: ≤ 48h after hospitalization/surgery;

*Pearson’s chi-square test.

The association between outcomes and nutritional risk groups was then tested. No difference was observed between the groups with regard to death and glycemic, potassium, and phosphorus disorders. However, the group classified as severe nutritional risk, presented a trend toward more severe sodium disorders (p = 0.054) and significantly more severe magnesium disorders (p = 0.007) (shown in Table 3).

**Table 3:** Association of outcomes in relation to nutritional risk by the NRS score, 2002 – Recife, PE, Brazil, 2022.

Outcomes	Moderate risk [n = 40]	Severe risk [n = 44]	p*
Clinical Outcome			
Discharge	26 [65%]	29 [65.9%]	0.930
Death	14 [35%]	15 [34.1%]	
Glucose disorder			
No	8 [20%]	4 [9.1%]	0.154
Yes	32 [80%]	40 [90.9%]	
Sodium disorder			
No	18 [45%]	11 [25%]	0.054
Yes	22 [55%]	33 [75%]	
Potassium disorder			
No	15 [37.5%]	14 [31.8%]	0.584
Yes	25 [62.5%]	30 [68.2%]	
Phosphorus disorder			
No	15 [37.5%]	16 [36.4%]	0.914
Yes	25 [62.5%]	28 [63.6%]	
Magnesium disorder			
No	29 [72.5%]	19 [43.2%]	0.007
Yes	11 [27.5%]	25 [56.8%]	

*Pearson’s chi-square test.

Lastly, the relative risk of death and metabolic disorders was analyzed per nutritional risk group and continuous risk (at each 1-point increase in the score). No association was observed between nutritional risk (stratified by group or continuous) and the risk of death or glucose, potassium or phosphorus disorders. The RR (CI 95%), however, adjusted for BMI, type of access, and length of stay for sodium disorders, was of 4.88 (1.5–16.2), being significantly higher in the severe risk group (p = 0.009), although this difference was not observed in the continuous nutritional risk analysis, of 1.4 (0.84–2.40) (p = 0.196). The severe nutritional risk group presented a relative risk of magnesium disorders (unadjusted and adjusted) of 1.75 (1.16–2.65) (p = 0.007) and 7.58 (2.33–24.6) (p = 0.001), respectively. Likewise, it was also observed that for every 1-point increase in nutritional risk, the relative risk of magnesium disorders (unadjusted and adjusted) was 1.78 (1.13–2.81) (p = 0.013) and 2.38 (1.34–4.24) (p = 0.003), respectively (shown in Table 4).

**Table 4:** Association of outcomes in relation to the nutritional risk group by the NRS, 2002 – Recife, PE, Brazil, 2022.

Nutritional risk group	Death		Glucose disorder		Sodium disorder		Potassium disorder		Phosphorus disorder		Magnesium disorder	
	RR [IC 95%]	p-value	RR [IC 95%]	p-value	RR [IC 95%]	p-value	RR [IC 95%]	p-value	RR [IC 95%]	p-value	RR [IC 95%]	p- value
**No Adjustment**												
Moderate	1.0		1.0		1.0		1.0		1.0		1.0	
Severe	0.97 [0.54–1.76]	0.930	1.67 [0.73–3.80]	0.54	1.58 [0.95–2.64]	0.054	1.12 [0.72–1.77]	0.584	1.02 [0.69–1.57]	0.914	**1.75** **[1.16–2.65]**	**0.007**
Adjusted*												
Moderate	1.0		1.0		1.0		1.0		1.0		1.0	
Severe	1.69 [0.58–4.92]	0.339	2.98 [0.73–12.2]	0.130	**4.88** **[1.5–16.2]**	**0.009**	1.88 [0.69–5.31]	0.231	1.34 [0.50–3.61]	0.554	**7.58** **[2.33–24.6]**	**0.001**
Continuous Risk**												
**No Adjustment**	1.19 [0.77–1.85]	0.424	1.23 [0.67–2.24]	0.496	1.12 [0.73–1.73]	0.604	1.12 [0.73–1.73]	0.604	1.10 [0.72–1.69]	0.654	**1.78** **[1.13–2.81]**	**0.013**
Adjusted*	1.48 [0.86–2.54]	0.156	1.28 [0.65–2.51]	0.468	1.40 [0.84–2.40]	0.196	1.29 [0.78–2.15]	0.324	1.18 [0.73–1.93]	0.497	**2.38** **[1.34–4.24]**	**0.003**

*Adjusted for BMI, type of access and length of stay

** Each 1 point increase in the score.

## DISCUSSION

The results of the NRS-2002 screening demonstrated a higher prevalence of a score equal to 5, which may be explained mainly by the patients’ nutritional status (score 3 = 53.6%) severity, and a median BMI = 20.4 kg/m^2^. It should be mentioned that the BMI cutoff point for the first screening stage of the NRS-2002 is 20.5 kg/m^2^, which is higher than the median observed, and that 52.4% were older patients, and for this population, the BMI levels of between 22 and 27 kg/m^2^ are considered eutrophic, which confirms the impact of nutritional status on the composition of the score^(22)^. The analysis of the association between the cofactors and the nutritional risk groups (moderate/severe) revealed that the groups were similar in relation to most of the variables analyzed. The only variable presenting a significant difference was the BMI (p = 0.001), which was an expected result since it is already part of the composition of the NRS-2002 score.

The association between the investigated metabolic changes and the nutritional risk groups demonstrated a trend towards more severe sodium disorders in the severe risk group (p = 0.054), as well as an adjusted relative risk of 4.88 (1.5–16.2) (p = 0.009) for developing these disorders. However, this difference was not verified by the continuous nutritional risk analysis. Sodium disorders, such as hyponatremia, are the most common electrolyte disorders and are associated with higher morbidity and mortality rates, especially in hospitalized patients and those on PN, due to the increased supply of liquids inherent to the therapy itself^(23)^.

A multicenter prospective study with non-critical patients receiving PN reported that severe malnutrition was the main risk factor for developing hyponatremia in patients on PN and is an independent factor for developing this disorder^(24,25)^. In addition to the poor nutritional status observed in the severe risk group, the study population was mainly represented by older patients (52.4%) and a high frequency of nutritional problems in this population, especially protein malnutrition, which also predisposes to the development of hyponatremia^(26)^. Another hypothesis proposed is that sarcopenia, which is associated with a reduction in muscle mass and, consequently, in the body potassium content, according to the Edelman-Boling equation, may induce hyponatremia, which could explain the frequency of this disorder usually observed in malnourished and older patients with sarcopenia^(27)^.

The severe nutritional risk group presented more severe magnesium disorders, which also occurred proportionally to the increase of each point in the NRS-2002 score. This electrolyte plays a key role in all enzymes involved in ATP synthesis, energy production and, indirectly, in muscle contraction and relaxation. Its deficiency is associated with poor muscle performance^(28)^. It is a fact that the domains related to nutritional status, disease severity and age, used to identify nutritional risk by the NRS-2002, are associated with magnesium disorders. Hypermagnesemia is rare; however, hypomagnesemia is more frequent in older patients^(29)^, and occurs in 12% of hospitalized patients, affecting 60–65% of those in intensive care, and may result from gastrointestinal (dietary restriction, diarrhea, poor absorption, pancreatitis, chronic use of proton inhibitors or genetic causes) or urinary losses due to the use of drugs such as thiazides^(30)^. As mentioned above, our population consisted mainly of older patients, and this group, in addition to a higher nutritional risk, presented with lower magnesium intake and intestinal absorption, as well as a greater renal excretion of magnesium^(31)^. A cross-sectional study assessed the prevalence of hypomagnesemia in older patients in a tertiary care center, where it was observed that 45% had some magnesium disorder, and of these, 29% presented hypomagnesemia^(31)^.

This study has presented some limitations, including the difficulty in expanding the number of patients in the study due to interrupted collection during critical periods of the COVID-19 pandemic, which may have influenced the significance of the results and limited the exploration of certain data. Another limitation was the use of a convenience sample, especially since the study was conducted during the pandemic. However, the multivariate adjustment of the statistical analysis minimizes a possible selection bias. One aspect that may restrain more evident associations in the two groups studied is the homogeneity of the sample, including the fact that groups without a risk < 3 and with a nutritional risk ≥ 3 were not compared according to the NRS-2002.

These results reinforce the need for adequate monitoring, not only of plasma magnesium levels, but of the clinical conditions that may lead to a deficiency of this nutrient and the need to use effective strategies for supplementation and adequacy of intake through PN, especially in the geriatric population.

The participation of the multidisciplinary nutritional therapy team, especially doctors, nurses, nutritionists and pharmacists, is essential, from nutritional risk screening, daily metabolic and clinical monitoring to the discontinuation of parenteral nutrition, to minimize the risk of complications and provide a better prognosis for the patient. It is important to note that in some institutions, this nutritional screening is initially carried out by the nurse, who will refer the patient to the dietician or other multidisciplinary team professionals in cases where nutritional risk is identified. This strategy can mitigate the risk of developing or worsening malnutrition in hospitalized patients, since, in most cases, the nurse is the first professional to receive the patient, thus facilitating the implementation of measures for early nutritional therapy.

## CONCLUSION

The stratification of the NRS 2002 nutritional risk range into groups (moderate/severe), in patients with PN, demonstrated that patients allocated to the severe risk group had a greater risk of developing greater sodium and magnesium metabolic disorders, suggesting that the interpretation of the NRS 2002 score currently used may underestimate the risk of developing more serious metabolic and clinical complications. The use of this stratification proposal identifies, among patients who are at nutritional risk, those who deserve even more specialized and individualized nutritional care, with the involvement of all members of the Multidisciplinary Nutritional Therapy Team. In addition, nutritional support (oral, enteral or parenteral) can be implemented earlier and more assertively to prevent the development or worsening of malnutrition in the hospital environment in adult patients and especially in the elderly.
