# Supplementary material for: Nutrition risk stratification and metabolic disorders in patients on parenteral nutrition
Source: Rev Esc Enferm USP. 2025 Apr 11;59:e20240274. doi: 10.1590/1980-220X-REEUSP-2024-0274en (PMC11991668; doi:10.1590/1980-220X-REEUSP-2024-0274en)
Supplement: Translation

# Translation: Estratificación del riesgo nutricional y de los trastornos metabólicos en pacientes en nutrición parenteral

**DOI:** 10.1590/1980-220X-REEUSP-2024-0274es

**Authors:** Rodrigo Luis da Silveira Silva, Ranna Rayssa Leal dos Santos, Helder Cardoso Tavares, Ulisses Ramos Montarroyos, Ana Célia Oliveira dos Santos

**Affiliations:** 1 Universidade de Pernambuco, Faculdade de Ciências Médicas, Recife, PE, Brasil.; 2 Universidade de Pernambuco, Instituto de Ciências Biológicas, Recife, PE, Brasil.; 3 Universidade de Pernambuco, Hospital Universitário Oswaldo Cruz, Recife, PE, Brasil.

**Keywords:** Evaluación Nutricional, Nutrición, Parenteral, Sodio, Magnesio

## Abstract

**Objetivo:**

Evaluar la asociación entre trastornos metabólicos y muerte en pacientes que reciben Nutrición Parenteral (NP), según una propuesta de estratificación en grupos de riesgo nutricional, utilizando el Nutritional Risk Screening, 2002 (NRS-2002).

**Método:**

Cohorte de 84 pacientes que recibían NP, con una puntuación NRS-2002 ≥ 3, clasificados en dos subgrupos: riesgo moderado (NRS 3-4) y riesgo grave (NRS 5-7). Se utilizaron datos secundarios de registros médicos y nutricionales. Las variables de resultado fueron “presencia de trastornos metabólicos y muerte” y las variables de exposición, “riesgo nutricional moderado y grave”. Se realizaron análisis descriptivos y bivariados y modelos de regresión logística.

**Resultados:**

No se observaron diferencias entre los grupos en cuanto a muerte. El RR ajustado (IC 95%) para los trastornos del sodio, 4,88 (1,5–16,2) (p = 0,009), y para los trastornos del magnesio, 7,58 (2,33–24,6) (p = 0,001), fue mayor en el grupo de riesgo grave.

**Conclusión:**

La estratificación del rango de riesgo nutricional en riesgo moderado y grave permitió identificar a los pacientes con mayor riesgo de desarrollar trastornos metabólicos, especialmente de sodio y magnesio.

## INTRODUCCIÓN

Los pacientes hospitalizados pueden presentar desnutrición en el momento del ingreso o desarrollarla durante la hospitalización^(1)^. En los países de América Latina, entre el 40 y el 60% de los pacientes adultos hospitalizados presentan desnutrición en el momento del ingreso^(2)^. Esta alta prevalencia se asocia con estancias hospitalarias más prolongadas, complicaciones graves (lesiones por presión, infecciones, caídas), una mayor frecuencia de reingresos y costes hospitalarios, además de mayores tasas de mortalidad^(2,3)^. Existen varias herramientas validadas que pueden utilizarse para la detección del riesgo nutricional con el fin de estimar la probabilidad de un mejor o peor pronóstico a través de los factores de riesgo asociados a la desnutrición^(4)^.

El Nutritional Risk Screening 2002 (NRS 2002) es una de las herramientas más utilizadas en la práctica clínica, se lleva a cabo mediante un proceso rápido y sencillo, dirigido por el equipo de admisión del hospital (médico, enfermero o dietista), incluye dominios relacionados con el estado nutricional y la gravedad de la enfermedad, y añade un punto extra si el paciente es mayor de 70 años^(5)^. La puntuación del estado nutricional (PEN) evalúa principalmente el porcentaje de pérdida de peso a lo largo del tiempo, el índice de masa corporal (IMC) y la reciente reducción del consumo de alimentos. La puntuación de la gravedad de la enfermedad (PGE) se basa en el impacto que un diagnóstico o una afección clínica causan en las necesidades nutricionales de un paciente^(5)^.

Se ha informado de que los pacientes con riesgo nutricional (NRS ≥ 3) tienen estancias hospitalarias más largas y presentan complicaciones quirúrgicas^(4,6)^, tasas de mortalidad más altas y más tempranas^(7)^ y mayores costes hospitalarios^(8)^ en comparación con los pacientes sin riesgo nutricional. Se ha demostrado que, en pacientes hospitalizados, por cada aumento de ١ punto en la puntuación NRS, se observa un aumento significativo de la mortalidad a los ٣٠ y ١٨٠ días, así como una estancia hospitalaria más larga (y un peor pronóstico funcional a los ١٨٠ días)^(9)^. Este resultado sugiere que una interpretación del resultado de la NRS 2002 a través de la clasificación actual (sin riesgo < 3 y con riesgo ≥ 3) puede subestimar la gravedad de la condición nutricional y sus complicaciones asociadas y también puede interferir con la definición del nivel de asistencia nutricional individualizada para cada paciente.

La nutrición parenteral (NP) es una terapia costosa y compleja, que se utiliza a menudo cuando los pacientes presentan afecciones clínicas asociadas a riesgo nutricional o desnutrición, lo que puede contribuir a la aparición de complicaciones metabólicas e infecciosas graves, a un aumento del tiempo de hospitalización y de la morbilidad cuando no se administra o prescribe adecuadamente y debe evaluarse y monitorearse continuamente^(10,11)^. Teniendo en cuenta que los pacientes en NP están desnutridos o en riesgo nutricional y que estas condiciones pueden agravar su pronóstico, este estudio ha tenido como objetivo evaluar la asociación entre el riesgo de trastornos metabólicos y la muerte, de acuerdo con una nueva propuesta de estratificación en grupos de riesgo nutricional (moderado y grave).

Cabe señalar que la nueva propuesta de estratificación no pretende cambiar los elementos que componen el NRS 2002, por lo que puede utilizarse con la misma población y alcance que el estudio original, y puede aplicarse a pacientes adultos y mayores hospitalizados, independientemente de la vía utilizada para la nutrición del paciente (oral, enteral o parenteral). Sin embargo, sugiere una nueva forma de interpretar los resultados, mejorando la aplicabilidad clínica de esta herramienta.

## MÉTODO

### Diseño, Periodo y Ubicación del Estudio

El presente estudio se describe de acuerdo con la lista de verificación de la declaración STROBE^(12)^. Se trata de un estudio de cohorte observacional retrospectivo realizado en un hospital universitario público, centro de referencia para enfermedades infecciosas y parasitarias, entre junio de 2020 y agosto de 2022.

### Población y Muestra: Criterios de Inclusión y Exclusión

La población estaba formada por 84 pacientes adultos que recibían nutrición parenteral total (NPT) y que estaban hospitalizados en el centro del estudio. La muestra fue por conveniencia y consistió en pacientes hospitalizados durante el período de estudio y que utilizaron NPT como vía exclusiva de nutrición. Los pacientes incluidos tenían ≥ 18 años, con riesgo nutricional (RN ≥ 3), que recibían NPT central o periférica, ingresados en las salas y unidades de cuidados intensivos. El NRS 2002 se llevó a cabo cuando los pacientes fueron admitidos para identificar el riesgo nutricional. El screening investiga inicialmente si el IMC es inferior a 20,5 kg/m^2^, si el paciente ha perdido peso en los últimos 3 meses, si ha habido una reducción en la ingesta de alimentos en la última semana o si el paciente está gravemente enfermo. Si alguna de las respuestas era afirmativa, el screening pasaba a la siguiente etapa, en la que se medía el deterioro del estado nutricional y la gravedad de la enfermedad o el grado de estrés. En función de los resultados obtenidos, el paciente se clasificaba como en riesgo nutricional si la puntuación era ≥ 3. Se excluía a los pacientes que recibían nutrición parenteral durante menos de 24 horas y a aquellos que presentaban alteraciones metabólicas o electrolíticas que no podían corregirse antes de iniciar la NP.

### Protocolo del Estudio

Los datos fueron recopilados por los responsables del estudio de los registros médicos del paciente, el formulario de seguimiento nutricional y el seguimiento clínico. Se recopilaron los siguientes datos: sexo, edad, presencia de comorbilidades, lugar de ingreso hospitalario, duración de la estancia hospitalaria, tiempo en días para iniciar la NP y tiempo total de uso, tipo de acceso venoso utilizado. La NP temprana se definió cuando su inicio se produjo en las 48 horas siguientes a la hospitalización del paciente, el evento quirúrgico o clínico que indicó la NP. Los resultados observados fueron la presencia de trastornos glucémicos y electrolíticos (Na, K, P y Mg) durante el uso exclusivo de la NP, y el resultado (muerte/alta) hasta el final de la NP. No hubo seguimiento para evaluar la recurrencia de trastornos metabólicos después del alta de los pacientes. Se realizaron pruebas de laboratorio a diario y se solicitaron según el protocolo establecido en el hospital, adaptado según Worthington et al.^(13)^.

Entre los pacientes incluidos en el estudio, basándose en la evaluación del riesgo nutricional (NRS 2002), se formaron 2 subgrupos: riesgo moderado (puntuación 3-4) y riesgo grave (puntuación 5-7). El razonamiento utilizado para estratificar los grupos se basó en lo que ya se ha demostrado en la literatura, que por cada aumento de 1 punto en la puntuación de riesgo nutricional, aumenta el riesgo de desarrollar complicaciones. Por lo tanto, decidimos dividir el rango de riesgo nutricional (puntuación de 3 a 7) en dos estratos (3-4) y (5-7).

Se tomaron medidas antropométricas para identificar el peso y la altura con el fin de calcular el índice de masa corporal (IMC), uno de los criterios utilizados en la fase inicial del NRS 2002. Se midieron la altura y el peso actuales de los pacientes que podían caminar, y las medidas de los que no podían caminar se estimaron según Chumlea et al.^(14,15)^. Para estimar las necesidades nutricionales, se utilizó el peso medido en la evaluación antropométrica; sin embargo, en pacientes obesos, se utilizó el peso ajustado^(16)^, y para aquellos con anasarca, se utilizó el peso promedio según el índice de masa corporal (IMC) para adultos y personas mayores^(17,18)^.

Los pacientes se clasificaron, según el protocolo del hospital, en críticos si estaban hospitalizados en una unidad de cuidados intensivos, con o en riesgo de desarrollar insuficiencia de órganos vitales, y necesitaban vigilancia continua y soporte vital (terapia ventilatoria, hemodinámica o de reemplazo renal). Los pacientes se consideraron estables si ingresaron en la sala sin necesidad de vigilancia continua o soporte ventilatorio o hemodinámico. Las indicaciones para el uso de NP siguieron las directrices de la ASPEN^(13)^.

Los trastornos de glucosa y electrolitos se consideraron como la presencia de niveles plasmáticos por encima o por debajo del rango de referencia utilizado por el laboratorio de la institución: glucemia: pacientes estables (60–99 mg/dl) y pacientes críticamente enfermos (60–180 mg/dl); sodio (135–148 mEq/l); potasio (3,5–5,3 mEq/l); fósforo (2,7–4,5 mg/dl); magnesio (1,46–2,2 mg/dl); AST (10–50 U/l); ALT (10–50 U/l).

La ingesta de proteínas y calorías de los pacientes estables se consideró adecuada cuando se encontraba dentro del rango de 1,2 a 2,0 g de proteína/kg/día y de 25 a 35 kcal/kg/día, respectivamente^(19,20)^. En pacientes en estado crítico, se consideró adecuada una ingesta de proteínas de 1,5 a 2,0 g de proteína/kg/día y una ingesta calórica de 15 a 20 Kcal/kg/día hasta el 4.º día de NP, y de ٢٥ a ٣٠ Kcal/kg/día a partir del ٥.º día en pacientes en fase de recuperación^(20)^. Se calculó el porcentaje de adecuación calórica y proteica en relación con los objetivos establecidos, considerando adecuada una ingesta calórica y proteica diaria de al menos el 70% de las necesidades nutricionales estimadas.

La NP se prescribió teniendo en cuenta el diagnóstico clínico y nutricional de cada paciente, respetando los límites establecidos en las pautas de terapia nutricional para líquidos, macronutrientes y micronutrientes, a fin de no inducir alteraciones metabólicas causadas por una administración inadecuada de nutrientes: glucosa (4-5 mg/kg/min), lípidos (<1 g/kg/día), líquidos (30–40 ml/día), calcio (10–15 mEq/día), magnesio (8–20 mEq/día), fósforo (20–40 mmol/día), sodio (1–2 mEq/kg/día), potasio (1–2 mEq/kg/día), así como las dosis diarias recomendadas de vitaminas y oligoelementos^(21)^.

Todos los pacientes recibieron bolsas de NP individualizadas, recetadas y monitoreadas por un equipo multidisciplinario de médicos, dietistas registrados, enfermeras y farmacéuticos. Las bolsas de NP se manipularon de acuerdo con la prescripción nutricional diaria y se administraron en un plazo de 24 horas a través de una bomba de infusión continua. Para la evolución del comportamiento nutricional, se aplicó el protocolo establecido por la institución, en el que los pacientes comenzaban con el 30% de sus necesidades nutricionales estimadas (con al menos 100 g de glucosa al día), progresando hasta el 50%, el 70% y alcanzando el 100% después del quinto día, si el paciente no presentaba complicaciones.

### Análisis de Resultados y Estadísticas

Los datos se analizaron utilizando el STATA IC 14,2 (STATA Corporation). Las variables categóricas se describieron en medidas de frecuencia absoluta y relativa, mientras que las variables numéricas se describieron en rangos de mediana e intercuartílicos (P_25_–P_75_), ya que no presentaban una distribución normal. Para probar la asociación entre variables categóricas binarias, se utilizó la prueba de chi-cuadrado de Pearson (2×2). Se aplicó un análisis de regresión logística para estimar el riesgo relativo (RR) y los respectivos intervalos de confianza (IC del 95%) en la asociación de los resultados con los grupos de riesgo nutricional. Para ajustar los factores de confusión, el criterio adoptado fue una significación de hasta el 10% (p < 0,1) en el análisis bivariado con riesgo nutricional. De acuerdo con este criterio, el RR se ajustó para el IMC, el tipo de acceso y la duración de la estancia. El RR se presentó con y sin ajuste multivariante para mostrar el efecto de estas variables en la asociación entre el riesgo nutricional y los resultados. La significación estadística adoptada fue del 5% para todos los análisis.

### Aspectos Éticos

El protocolo del estudio se llevó a cabo de acuerdo con las directrices éticas nacionales e internacionales y fue aprobado por el Comité de Ética de Investigación del Hospital Universitario Oswaldo Cruz (HUOC)/Pronto Socorro Cardiológico Universitario de Pernambuco Prof. Luiz Tavares (Complejo Hospitalario HUOC/PROCAPE), número de aprobación [4.432.953]. No se requirió el consentimiento del paciente porque se trataba de una investigación retrospectiva que utilizaba datos que ya se habían recopilado de forma rutinaria en formularios de monitoreo nutricional, registros de pacientes o bases de datos electrónicas. Además, los datos se analizaron y presentaron de forma anónima y agregada, sin permitir la identificación de los participantes en la investigación.

## RESULTADOS

Inicialmente se reclutaron noventa pacientes, pero se excluyeron seis por recibir NP durante un período inferior a 24 horas debido a la muerte o a complicaciones clínicas, con lo que el total ascendió a 84 pacientes. No hubo otras pérdidas o exclusiones, ya que el otro criterio de exclusión fue la presencia de trastornos metabólicos o electrolíticos que no pudieron corregirse antes de iniciar la NP, lo cual es poco frecuente, ya que el servicio cuenta con un protocolo para corregir los trastornos metabólicos y electrolíticos antes de iniciar la NP.

La población del estudio presentó una duración media de la estancia hospitalaria de 21,5 días y estuvo representada en su mayoría por hombres mayores, hospitalizados en salas quirúrgicas (67,9%), sin comorbilidades (52,4%), con una edad media de 61,5 años y un IMC de 20,4 kg/m^2^. La NP se administró principalmente a través de un acceso venoso central (79,8%) y la duración media de la NP fue de 9 días. Además, también se observó que el 71,4% de los pacientes iniciaron la nutrición parenteral de forma temprana. Más del 80% de los pacientes presentaban una adecuación calórica y proteica superior al 70% de las necesidades nutricionales estimadas. A través del NRS 2002, se verificó que la mayoría de los pacientes presentaban una puntuación de riesgo nutricional igual a 5 (42,9%) y que este resultado se componía principalmente de un agotamiento grave del estado nutricional de los pacientes (puntuación 3 = 53,6%) y una gravedad moderada de la enfermedad (puntuación 2 = 65,5%, como se muestra en la Tabla 1).

**Tabla 1:** Características de los pacientes que reciben nutrición parenteral – Recife, PE, Brasil, 2022.

Característica	n [%]/mediana/media
**Sexo**	
Masculino	44 [52,4%]
**Rango de edad**	
Adulto (< 60 años)	40 [47,6%]
Adulto mayor (≥ 60 años)	44 [52,4%]
**IMC (kg/m^2^)**	20,4 [17,9–26,9]
**Con comorbilidades**	40 [47,6%]
**Lugar de estancia en el hospital**	
UCI	16 [19,0%]
Sala médica	11 [13,1%]
Sala quirúrgica	67 [67,9%]
**Tipo de acceso venoso**	
Periférico	17 [20,2%]
Central	67 [79,8%]
**Días de hospitalización**	21,5 [13–32]
**Días con NP**	9 [6–18]
**NP temprana**	60 [71,4%]
**Composición de la puntuación NRS 2002**	
***Estado nutricional***	
Puntuación 0	8 [9,5%]
Puntuación 1	11 [13,1%]
Puntuación 2	20 [23,8%]
Puntuación 3	45 [53,6%]
***Severidad de la enfermedad***	
Puntuación 0	1 [1,2%]
Puntuación 1	15 [17,9%]
Puntuación 2	55 [65,5%]
Puntuación 3	13 [15,5%]
***Edad (> 70 años)***	
Puntuación 0	68 [81,0%]
Puntuación 1	16 [19,0%]
**Calorías adecuadas**	92,5 [±14,9]
> 70%	74 [88,1%]
≤ 70%	10 [11,9%]
**Proteína adecuada**	93,8 [±14,1]
> 70%	76 [90,5%]
≤ 70%	8 [9,5%]
**Trastornos metabólicos**	
**Glucosa**	72 [85,7%]
**Sodio**	55 [65,5%]
**Potasio**	55 [65,5%]
**Fósforo**	53 [63,1%]
**Magnesio**	36 [42,9%]

IMC: Índice de masa corporal; NP: Nutrición parenteral; NRS 2002 Nutritional Risk Screening: Evaluación de riesgos nutricionales 2002; Temprana: ≤ 48 h después de la hospitalización/cirugía.

Para verificar si existía una diferencia entre los grupos de riesgo moderado y grave, se estableció inicialmente una asociación entre los cofactores analizados y los respectivos grupos. No se observaron diferencias entre los grupos en la mayoría de las variables, salvo en el IMC, que fue menor en el grupo de riesgo nutricional grave (p = 0,001) (como se muestra en la Tabla 2).

**Tabla 2:** Asociación de variables analizadas en relación con el riesgo nutricional según la puntuación del NRS, 2002 – Recife, PE, Brasil, 2022.

Variables	Riesgo moderado [n = 40]	Riesgo grave [n = 44]	p*
Sexo			
Femenino	18 [45%]	22 [50%]	0,647
Masculino	22 [55%]	22 [50%]	
IMC (kg/m^2^)	24,5 [19,9–28,7]	18,7 [16,2–22,5]	0,001
Comorbilidades			
No	22 [55%]	22 [50%]	0,647
Si	18 [45%]	22 [50%]	
Tipo de acceso venoso			
Periférico	5 [12,5%]	12 [27,3%]	0,092
Central	35 [87,5%]	32 [72,7%]	
Días de hospitalización			
≤ 21 días	16 [40%]	26 [59,1%]	0,081
> 21 días	24 [60%]	18 [40,9%]	
Días en NP			
≤ 9 días	18 [45%]	26 [59,1%]	0,197
> 9 días	22 [55%]	18 [40,9%]	
NP temprana			
No	11 [27,5%]	13 [29,5%]	0,836
Si	29 [72,5%]	31 [70,5%]	
Calorías adecuadas			
> 70%	6 [15%]	4 [9,1%]	0,404
≤ 70%	34 [85%]	40 [90,9%]	
Proteínas adecuadas			
> 70%	4 [10%]	4 [9,1%]	0,887
≤ 70%	36 [90%]	40 [90,9%]	

IMC: índice de masa corporal; NP: nutrición parenteral; NRS 2002: Nutritional Risk Screening 2002 (evaluación del riesgo nutricional de 2002); Temprano: ≤ 48 h después de la hospitalización/cirugía

*prueba de chi-cuadrado de Pearson.

A continuación, se analizó la relación entre los resultados y los grupos de riesgo nutricional. No se observaron diferencias entre los grupos en cuanto a muerte y trastornos glicémicos, de potasio y de fósforo. Sin embargo, el grupo clasificado como de riesgo nutricional grave presentó una tendencia hacia trastornos de sodio más graves (p = 0,054) y trastornos de magnesio significativamente más graves (p = 0,007) (como se muestra en la Tabla 3).

**Tabla 3:** Asociación de resultados en relación con el riesgo nutricional según la puntuación NRS, 2002 – Recife, PE, Brasil, 2022.

Resultados	Riesgo moderado [n = 40]	Riesgo grave [n = 44]	p*
Resultado Clínico			
Alta	26 [65%]	29 [65,9%]	0,930
Muerte	14 [35%]	15 [34,1%]	
Alteración de Glucosa			
No	8 [20%]	4 [9,1%]	0,154
Si	32 [80%]	40 [90,9%]	
Alteración de Sodio			
No	18 [45%]	11 [25%]	0,054
Si	22 [55%]	33 [75%]	
Alteración de Potasio			
No	15 [37,5%]	14 [31,8%]	0,584
Si	25 [62,5%]	30 [68,2%]	
Alteración de Fósforo			
No	15 [37,5%]	16 [36,4%]	0,914
Si	25 [62,5%]	28 [63,6%]	
Alteración de Magnesio			
No	29 [72,5%]	19 [43,2%]	0,007
Si	11 [27,5%]	25 [56,8%]	

*prueba de chi-cuadrado de Pearson.

Por último, se analizó el riesgo relativo de muerte y trastornos metabólicos por grupo de riesgo nutricional y riesgo continuo (a cada aumento de 1 punto en la puntuación). No se observó asociación entre el riesgo nutricional (estratificado por grupo o continuo) y el riesgo de muerte o de trastornos de glucosa, potasio o fósforo. Sin embargo, el RR (IC 95%), ajustado por IMC, tipo de acceso y duración de la estancia para los trastornos de sodio, fue de 4,88 (1,5–16,2), siendo significativamente mayor en el grupo de riesgo grave (p = 0,009), aunque esta diferencia no se observó en el análisis de riesgo nutricional continuo, de 1,4 (0,7-2,7). 88 (1,5–16,2), siendo significativamente mayor en el grupo de riesgo grave (p = 0,009), aunque esta diferencia no se observó en el análisis de riesgo nutricional continuo, de 1,4 (0,84–2,40) (p = 0,196). El grupo de riesgo nutricional grave presentó un riesgo relativo de trastornos de magnesio (sin ajustar y ajustado) de 1,75 (1,16–2,65) (p = 0,007) y 7,58 (2,33–24,6) (p = 0,001), respectivamente. Asimismo, también se observó que, por cada aumento de 1 punto en el riesgo nutricional, el riesgo relativo de trastornos de magnesio (sin ajustar y ajustado) era de 1,78 (1,13–2,81) (p = 0,013) y 2,38 (1,34–4,24) (p = 0,003), respectivamente (como se muestra en la Tabla 4).

**Tabla 4:** Asociación de resultados en relación con el grupo de riesgo nutricional según el NRS, 2002 – Recife, PE, Brasil, 2022.

Grupo de riesgo nutricional	Muerte		Alteración de glucosa		Alteración de sodio		Alteración de potasio		Alteración de fósforo		Alteración de magnesio	
	RR [IC 95%]	p-valor	RR [IC 95%]	p-valor	RR [IC 95%]	p-valor	RR [IC 95%]	p-valor	RR [IC 95%]	p-valor	RR [IC 95%]	p- valor
**Sin ajuste**												
Moderado	1,0		1,0		1,0		1,0		1,0		1,0	
Grave	0,97[0,54–1,76]	0,930	1,67[0,73–3,80]	0,54	1,58[0,95–2,64]	0,054	1,12[0,72–1,77]	0,584	1,02[0,69–1,57]	0,914	**1,75** **[1,16–2,65]**	**0,007**
Ajustado*												
Moderado	1,0		1,0		1,0		1,0		1,0		1,0	
Grave	1,69[0,58–4,92]	0,339	2,98[0,73–12,2]	0,130	**4,88** **[1,5–16,2]**	**0,009**	1,88[0,69–5,31]	0,231	1,34[0,50–3,61]	0,554	**7,5** **8[2,33–24,6]**	**0,001**
Riesgo continuo**												
**Sin ajuste**	1,19[0,77–1,85]	0,424	1,23[0,67–2,24]	0,496	1,12[0,73–1,73]	0,604	1,12[0,73–1,73]	0,604	1,10[0,72–1,69]	0,654	**1,78** **[1,13–2,81]**	**0,013**
Ajustado*	1,48[0,86–2,54]	0,156	1,28[0,65–2,51]	0,468	1,40[0,84–2,40]	0,196	1,29[0,78–2,15]	0,324	1,18[0,73–1,93]	0,497	**2,38** **[1,34–4,24]**	**0,003**

*Ajustado por IMC, tipo de acceso y duración de la estadía

**Cada aumento de 1 punto en la puntuación.

## DISCUSIÓN

Los resultados del screening NRS-2002 demostraron una mayor prevalencia de una puntuación igual a 5, lo que puede explicarse principalmente por el estado nutricional de los pacientes (puntuación 3 = 53,6%) y una mediana de IMC = 20,4 kg/m^2^. Cabe mencionar que el punto de corte del IMC para la primera etapa de screening del NRS-2002 es de 20,5 kg/m^2^, que es superior a la mediana observada, y que el 52,4% eran pacientes de edad avanzada, y para esta población, los niveles de IMC de entre 22 y 27 kg/m^2^ se consideran eutróficos, lo que confirma el impacto del estado nutricional en la composición de la puntuación^(22)^. El análisis de la asociación entre los cofactores y los grupos de riesgo nutricional (moderado/grave) reveló que los grupos eran similares en relación con la mayoría de las variables analizadas. La única variable que presentaba una diferencia significativa era el IMC (p = 0,001), lo cual era un resultado esperado, ya que forma parte de la composición de la puntuación NRS-2002.

La asociación entre los cambios metabólicos investigados y los grupos de riesgo nutricional demostró una tendencia hacia trastornos de sodio más graves en el grupo de riesgo grave (p = 0,054), así como un riesgo relativo ajustado de 4,88 (1,5–16,2) (p = 0,009) para desarrollar estos trastornos. Sin embargo, esta diferencia no fue verificada por el análisis continuo de riesgo nutricional. Los trastornos del sodio, como la hiponatremia, son los trastornos electrolíticos más comunes y se asocian con mayores tasas de morbilidad y mortalidad, especialmente en pacientes hospitalizados y en aquellos que reciben NP, debido al aumento del suministro de líquidos inherente a la terapia en sí^(23)^.

Un estudio prospectivo multicéntrico con pacientes no críticos que recibían NP informó de que la desnutrición grave era el principal factor de riesgo de desarrollar hiponatremia en pacientes en NP y es un factor independiente para desarrollar este trastorno^(24,25)^. Además del mal estado nutricional observado en el grupo de riesgo grave, la población del estudio estaba representada principalmente por pacientes de edad avanzada (52,4%) y una alta frecuencia de problemas nutricionales en esta población, especialmente desnutrición proteica, que también predispone al desarrollo de hiponatremia^(26)^. Otra hipótesis propuesta es que la sarcopenia, que se asocia con una reducción de la masa muscular y, en consecuencia, del contenido corporal de potasio, según la ecuación de Edelman-Boling, puede inducir hiponatremia, lo que podría explicar la frecuencia de este trastorno que suele observarse en pacientes desnutridos y ancianos con sarcopenia^(27)^.

El grupo de riesgo nutricional grave presentó trastornos de magnesio más graves, que también se produjeron proporcionalmente al aumento de cada punto en la puntuación NRS-2002. Este electrolito desempeña un papel clave en todas las enzimas implicadas en la síntesis de ATP, la producción de energía e, indirectamente, en la contracción y relajación muscular. Su deficiencia se asocia a un bajo rendimiento muscular^(28)^. Es un hecho que los dominios relacionados con el estado nutricional, la gravedad de la enfermedad y la edad, utilizados para identificar el riesgo nutricional por el NRS-2002, están asociados a trastornos del magnesio. La hipermagnesemia es poco frecuente; sin embargo, la hipomagnesemia es más frecuente en pacientes de edad avanzada^(29)^, y se produce en el 12% de los pacientes hospitalizados, afectando al 60-65% de los que están en cuidados intensivos, y puede ser el resultado de pérdidas gastrointestinales (restricción dietética, diarrea, mala absorción, pancreatitis, uso crónico de inhibidores de protones o causas genéticas) o urinarias debido al uso de fármacos como las tiazidas^(30)^. Como se mencionó anteriormente, nuestra población consistía principalmente en pacientes mayores, y este grupo, además de un mayor riesgo nutricional, presentaba una menor ingesta de magnesio y absorción intestinal, así como una mayor excreción renal de magnesio^(31)^. Un estudio transversal evaluó la prevalencia de hipomagnesemia en pacientes mayores en un centro de atención terciaria, donde se observó que el 45% presentaba algún trastorno de magnesio, y de estos, el 29% presentaba hipomagnesemia^(31)^.

Este estudio ha presentado algunas limitaciones, entre ellas la dificultad para ampliar el número de pacientes en el estudio debido a la interrupción de la recogida de datos durante los períodos críticos de la pandemia de COVID-19, lo que puede haber influido en la importancia de los resultados y limitado la exploración de ciertos datos. Otra limitación fue el uso de una muestra de conveniencia, especialmente porque el estudio se llevó a cabo durante la pandemia. Sin embargo, el ajuste multivariante del análisis estadístico minimiza un posible sesgo de selección. Un aspecto que puede restringir las asociaciones más evidentes en los dos grupos estudiados es la homogeneidad de la muestra, incluido el hecho de que los grupos sin un riesgo < 3 y con un riesgo nutricional ≥ 3 no se compararon según la NRS-2002.

Estos resultados refuerzan la necesidad de un monitoreo adecuado, no solo de los niveles de magnesio en plasma, sino de las condiciones clínicas que pueden conducir a una deficiencia de este nutriente y la necesidad de utilizar estrategias efectivas para la suplementación y la adecuación de la ingesta a través de la NP, especialmente en la población geriátrica.

La participación del equipo multidisciplinario de terapia nutricional, especialmente médicos, enfermeras, nutricionistas y farmacéuticos, es esencial, desde la detección de riesgos nutricionales, el monitoreo metabólico y clínico diario hasta la interrupción de la nutrición parenteral, para minimizar el riesgo de complicaciones y proporcionar un mejor pronóstico para el paciente. Es importante señalar que, en algunas instituciones, este screening nutricional lo realiza inicialmente el personal de enfermería, que derivará al paciente al dietista u otros profesionales del equipo multidisciplinar en los casos en que se identifique un riesgo nutricional. Esta estrategia puede mitigar el riesgo de desarrollar o empeorar la malnutrición en pacientes hospitalizados, ya que, en la mayoría de los casos, el personal de enfermería es el primer profesional que recibe al paciente, lo que facilita la aplicación de medidas para una terapia nutricional temprana.

## CONCLUSIÓN

La estratificación del rango de riesgo nutricional NRS 2002 en grupos (moderado/grave), en pacientes con NP, demostró que los pacientes asignados al grupo de riesgo grave tenían un mayor riesgo de desarrollar trastornos metabólicos de sodio y magnesio más graves, lo que sugiere que la interpretación de la puntuación NRS 2002 utilizada actualmente puede subestimar el riesgo de desarrollar complicaciones metabólicas y clínicas más graves. El uso de esta propuesta de estratificación identifica, entre los pacientes que están en riesgo nutricional, a aquellos que merecen una atención nutricional aún más especializada e individualizada, con la participación de todos los miembros del Equipo Multidisciplinario de Terapia Nutricional. Además, el apoyo nutricional (oral, enteral o parenteral) puede implementarse antes y de manera más asertiva para prevenir el desarrollo o empeoramiento de la desnutrición en el entorno hospitalario en pacientes adultos y especialmente en ancianos.
